# Coronavirus Does It Again: Post-COVID-19 Hemophagocytic Lymphohistiocytosis (HLH)

**DOI:** 10.7759/cureus.35275

**Published:** 2023-02-21

**Authors:** Thanushiya Jeyakanthan, Luisa Ladel, Bhavna Khandpur, Wan Ying Tan, Syed Alishan Nasir

**Affiliations:** 1 Internal Medicine, Norwalk Hospital, Norwalk, USA; 2 Pathology, Norwalk Hospital, Norwalk, USA

**Keywords:** fever of unkown, multisystem inflammatory syndrome, post-covid sequelae, covid 19, secondary hlh

## Abstract

Hemophagocytic lymphohistiocytosis (HLH) is a hematological disorder that results from an uncontrolled activation of the immune system, which can then lead to multisystem organ failure. Given the nonspecific nature of this illness, it can go undetected for too long, thereby causing permanent damage to organ systems. In adults, HLH has been associated with a number of infectious etiologies, particularly viral infections. Coronavirus disease 2019 (COVID-19), caused by severe acute respiratory syndrome coronavirus 2 (SARS-CoV-2), has led to a global pandemic and has been associated with acute respiratory distress syndrome (ARDS). Among its other manifestations, COVID-19 has also been linked to HLH. In this report, we describe a case of a male patient who presented with multisystem organ failure and was found to have HLH. Since no clear etiology for his HLH could be elicited, it was determined to be a result of his recent COVID-19 infection.

## Introduction

Hemophagocytic lymphohistiocytosis (HLH) is an immune-mediated process characterized by an uncontrolled activation and disinhibition of cytotoxic T cells, macrophages, and natural killer (NK) cells [1]. While these cells normally function to target and destroy foreign entities in the body, in patients with HLH, predisposed gene mutations and nonspecific stimuli result in a generalized inflammatory response that causes global cytokine release, which can then lead to multiorgan destruction [1-2]. In adults, HLH can be a consequence of an impactful immunological state such as sepsis. Cases of HLH in adult patients have been identified in relation to coronavirus disease 2019 (COVID-19), caused by severe acute respiratory syndrome coronavirus 2 (SARS-CoV-2), likely from severe immune reaction mounted in response to this viral infection. Our report discusses a patient who was diagnosed with HLH after having recovered from a COVID-19 infection a month prior to admission.

## Case presentation

A 65-year-old male with a past medical history of recent COVID-19 infection about one month prior, treated with Paxlovid, presented to the emergency department with a four-day history of fevers up to 40.5 ℃ and a dry cough. On admission, the patient was noted to be febrile, normotensive, tachycardic, and tachypneic, requiring 4 L of oxygen via nasal cannula to maintain oxygen saturations greater than 92%. His physical exam was otherwise unremarkable. Laboratory studies on admission were significant for thrombocytopenia of 81,000/uL, acute kidney injury with a creatinine of 1.28 mg/dL and blood urea nitrogen (BUN) of 33 mg/dL (baseline creatinine below 1.0 mg/dL), mild transaminitis with aspartate aminotransferase (AST) of 90 U/L and alanine aminotransferase (ALT) of 79 U/L, as well as lactic acidosis with a lactic acid level of 4 mmol/L. Furthermore, his C-reactive protein (CRP) was elevated at 152 mg/L. Hemoglobin and WBC on admission were within normal limits at 16.3 g/dL and 4,700/uL, respectively. His chest X-ray was unremarkable. Respiratory viral panel testing for influenza, respiratory syncytial virus, and COVID-19 was negative.

**Figure 1:**
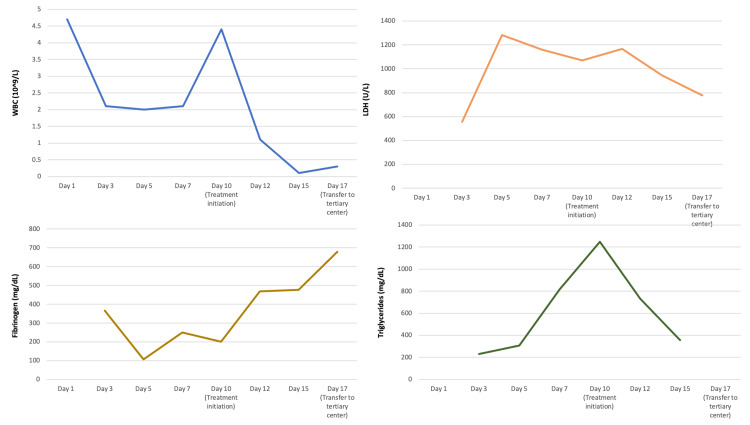
Graphs demonstrating pertinent laboratory value trends during the hospital course (A) White blood cell count (WBC), (B) lactate dehydrogenase (LDH), (C) fibrinogen level, and (C) triglycerides level. X-axis shows the days of hospital stay and Y-axis shows units of measurement of these lab values

Over the course of the hospital stay, the patient deteriorated clinically with acute hypoxemic respiratory failure requiring increased oxygen supplementation via a heated high-flow nasal cannula. He continued to have high-grade fevers up to 40.5 ℃ and developed a new productive cough with blood-tinged sputum. Repeat chest X-ray showed interval development of a right perihilar mass-like opacity. Despite the escalation of care and broadening of antibiotics, he continued to demonstrate signs of multisystem organ dysfunction with worsening thrombocytopenia, leukopenia, and renal dysfunction. Additional laboratory studies demonstrated an elevated lactate dehydrogenase (LDH) of 556 U/L, elevated ferritin of 25,570 ng/mL, new hypertriglyceridemia of 232 mg/dL as well as increasing transaminase levels with new conjugated hyperbilirubinemia of 2.3 mg/dL. Table 1 and Figure 1 show trends of the patient’s serum markers throughout his hospital stay.

**Table 1:** Laboratory trend throughout patient’s hospitalization WBC: white blood cell count; Hgb: hemoglobin; Plt: platelets; GFR: glomerular filtration rate; AST: aspartate transferase; ALT: alanine transaminase; TG: triglycerides; CK: creatine kinase; LDH: lactate dehydrogenase; INR: international normalized ratio

Lab values	WBC (10^9^/L)	Bands (%)	Hgb (g/dl)	Plt (10^9^/L)	GFR (ml/min/1.73m^2^)	AST (U/L)	ALT (U/L)	Total bilirubin (mg/dL)	Direct bilirubin (mg/dL)	Ferritin (ng/mL)	Fibrinogen (mg/dL)	TG (mg/dL)	CK (U/L)	LDH (U/L)	INR (ratio)
Day 1	4.7	10	16.3	81	56	79	90	1.1							
Day 3	2.1	16	14.1	44	46	173	299	1.6	1.3	25570	366	232		556	
Day 5	2	20	12.7	47	17	338	840	3.3	2.9	78620	107	307	387	1282	1.6
Day 7	2.1	24	11.7	31	9	443	1021	6	5.5	98261	249	817	483	1161	2.44
Day 10 (treatment initiation)	4.4	4	10	21	7	300	431	11.4	9.5	40085	201	1249	1342	1071	1.35
Day 12	1.1	1	9.2	22	8	200	208	12.5	11.7	17692	468	734	2560	1167	1.44
Day 15	0.1	0	7.4	10	16	256	262	14.5	13.1	8821	477	356		946	2.48
Day 17 (transfer to tertiary center)	0.3	0	7.9	19	15	223	120	16.7	14.5	7113	680		1358	779	2.14

**Figure 2:**
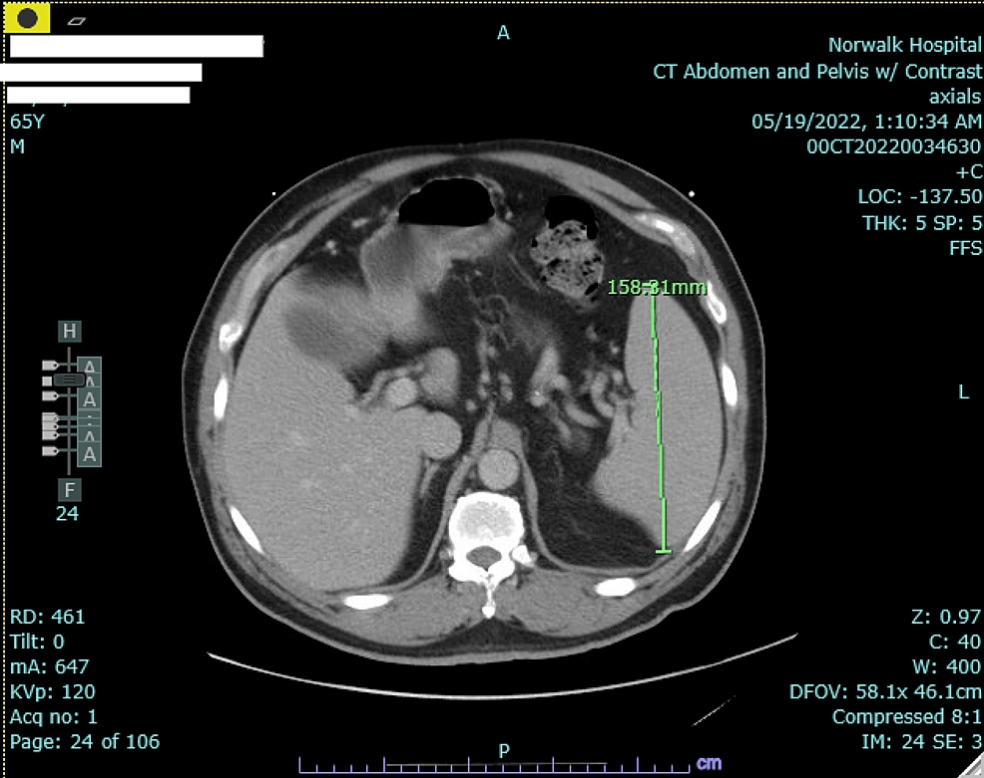
CT scan of the abdomen and pelvis with contrast shows splenomegaly measuring approximately 158.31 mm CT: computed tomography

The patient was initially started on IV antibiotics with ceftriaxone and azithromycin, and he was admitted for a severe systemic inflammatory syndrome of an unclear source. He underwent a CT scan (Figure 2) of the chest, abdomen, and pelvis, which revealed mild bronchial wall thickening and scattered small pulmonary nodules, splenomegaly, enlarged periaortic lymph nodes, and hepatic steatosis. Urine antigens for Legionella and Streptococcus pneumoniae were negative.

**Figure 3:**
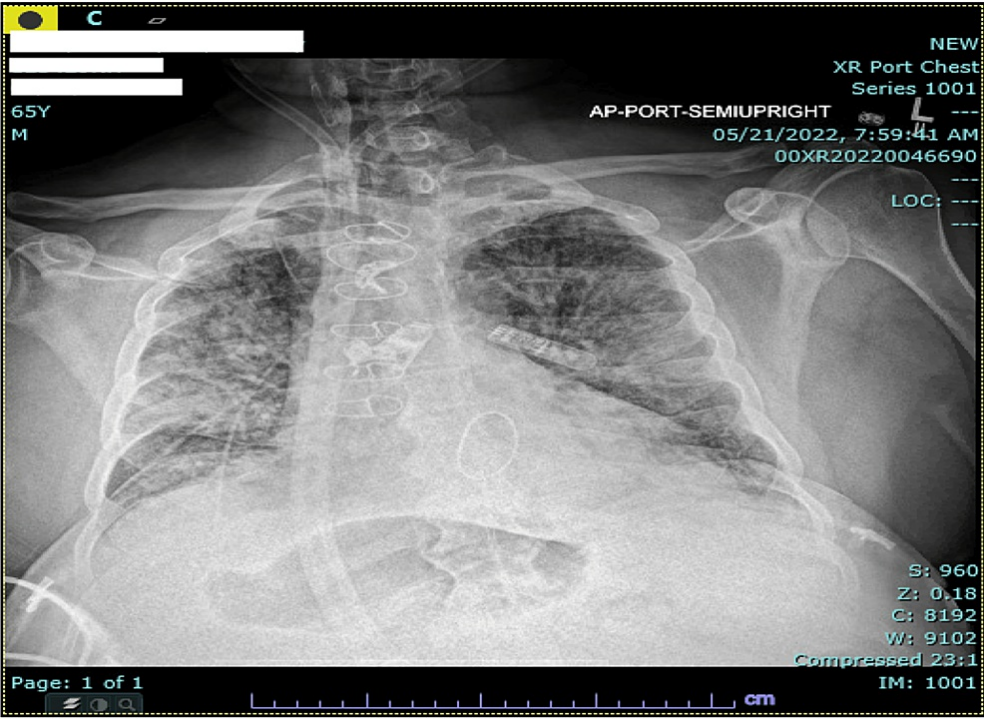
Portable chest X-ray prior to ICU transfer The image shows worsening fluffy airspace opacifications of bilateral lungs compatible with acute respiratory distress syndrome (ARDS)

The patient was subsequently transitioned to the ICU given findings of rapidly developing bilateral infiltrates consistent with acute respiratory distress syndrome (ARDS) on serial chest X-rays (Figure 3). Given his rapidly worsening respiratory status, he was intubated. At this time, pulse dose steroid therapy was initiated. Over the course of his hospital stay, extensive infectious workup was obtained, including a tick-borne panel (Lyme, Babesia, Anaplasma, Ehrlichia), hepatitis serologies, and HIV testing, which was negative. Multiple blood, urine, and sputum cultures showed no growth. Cytomegalovirus (CMV) and Epstein-Barr virus (EBV) IgMs were negative. We also pursued immunological workup with negative serum markers for antinuclear antibodies (ANA), antineutrophil cytoplasmic antibodies (ANCA), anti-Jo 1, ribosomal P protein antibody, RNP autoantibody, SCL-70, Smith autoantibody, SMA, and SSA.

**Figure 4:**
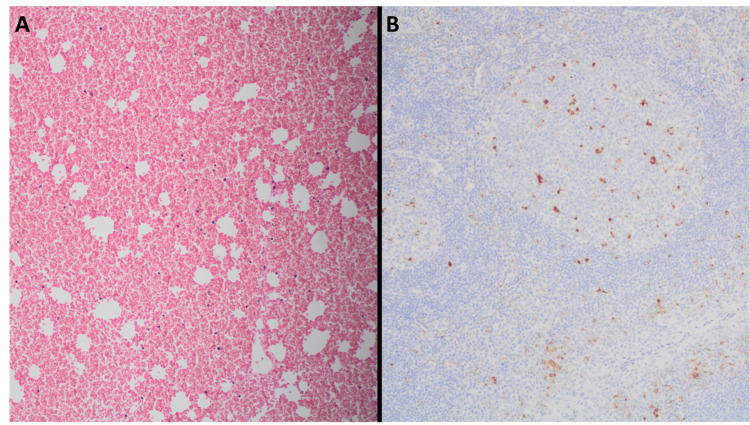
Bone marrow biopsy showing hypercellular bone marrow with histiocytes undergoing hemophagocytosis A: Hypercellular bone marrow - hematoxylin and eosin stain x100; B: CD68 stain showing histiocytes undergoing hemophagocytosis

Given the lack of improvement in the patient’s clinical status, negative infectious, rheumatological workup, and persistent multiorgan failure despite antibiotic therapy and supportive care, we suspected HLH. A bone marrow biopsy was performed (Figure 4), which revealed mature trilineage hematopoiesis with focal findings compatible with hemophagocytosis. No evidence was found for hematopoietic malignancy on histopathology or flow cytometry analysis.

With the biopsy findings as well as the patient’s splenomegaly, elevated ferritin levels, and new hypertriglyceridemia and pancytopenia, our patient fulfilled more than five out of eight criteria necessary for the diagnosis of HLH. Serum analysis performed a few days later showed a significant elevation of soluble CD25, which was consistent with HLH as well.

Once the diagnosis was established, all antibiotics were discontinued, and the patient was placed on guideline-based immuno-chemotherapy with dexamethasone and etoposide for HLH. The patient’s hospital course was unfortunately further complicated by tumor lysis syndrome, anuric acute tubular necrosis (ATN) requiring hemodialysis, persistent liver injury, disseminated intravascular coagulation (DIC), and severe thrombocytopenia refractory to platelet transfusion impeding treatment continuation with etoposide. In addition, the patient developed toxic metabolic encephalopathy as a result of his kidney and liver dysfunction, presenting a major barrier to extubation. A decision was made to transfer the patient to a tertiary care center for IL-2 receptor blocker therapy and evaluation of other viable treatment alternatives given his intractable thrombocytopenia.

## Discussion

An initial diagnosis of HLH is often difficult for clinicians to make given the nonspecific presentation of this disease process. At presentation, HLH can resemble sepsis with fever and multiorgan dysfunction, and hence an early diagnosis of this condition can be challenging. In the initial phase of sepsis, cytokines are released to activate white blood cells such as leukocytes, which encompass lymphocytes that are composed of T cells, B cells, NK cells, neutrophils, monocytes, and macrophages. Once the immune process has dealt with the pathogen, inhibitory chemical signals are generated in order to curb the ongoing immune response. However, in HLH, there is an uncontrolled release of cytokines, which activate lymphocytes to target self-antigens resulting in generalized organ dysfunction. HLH was first described in 1939 as a severe inflammatory reaction by our body’s innate immune cells targeted against self-hematopoietic lineages [2].

The diagnosis of HLH is based on the HLH-2004 diagnostic criteria proposed by the Histiocyte Society in 1991 and revised in 2004. Diagnosis is established if patients possess molecular evidence of a gene mutation consistent with HLH and/or meet five or more out of eight diagnostic criteria. The eight criteria include fever, splenomegaly, cytopenia affecting two or more hematopoietic cell lines (hemoglobin ≤9 g/dL, platelets <100000 L per μL, neutrophils <1000 cells per μL), elevated triglycerides levels (≥265 mg/dL) and/or low fibrinogen levels (≤150 mg/dL), signs of hemophagocytosis in the bone marrow, spleen, or lymph nodes without evidence of malignancy, low or absent NK cell cytotoxicity, elevated ferritin (≥500 ng/mL), and high levels of soluble CD25 (interleukin-2Rα chain ≥2400 IU/mL) [3-5].

HLH can be categorized as primary and secondary. Primary HLH is encountered in the pediatric population and is also known as familial hemophagocytic lymphohistiocytosis (FHL), which is caused by rare genetic mutations in certain genes that are passed down in an autosomal recessive inheritance pattern. There have been five different types identified: FHL1, FHL2, FHL3 FHL4, and FHL5, with genetic mutation coding of proteins in cytotoxic NK and T cells causing dysfunction [6-8]. These subtypes have identified and non-identified mutations in different loci of chromosomes performing various functions in the immune signaling cascade to destroy the cells without appropriate control or target. For instance, in FHL2, the PRF1 gene encoding for surface protein “perforin”, which normally functions as a passageway to send granzymes from NK/T cells to target cells to cause cell death, is mutated resulting in untriggered apoptosis [8]. Secondary or acquired HLH in the adult population is caused by triggering events such as infection (bacterial, viral, fungal, parasitic), autoimmune conditions, and malignancy without evidence of genetic defects. Various case reports and studies have described infection-associated hemophagocytic syndrome without any confounding factors. Viruses such as EBV (most common), other herpes viruses [human herpesvirus 8 (HHV8) and CMV], HIV, parvovirus, hepatitis virus, enterovirus, and influenza virus have been implicated in triggering reactive hemophagocytosis [9]. Since this is an immune-mediated process, management of both primary and secondary HLH includes immunosuppressants.

In recent years, since the start of the COVID-19 pandemic, cases of SARS-CoV-2-induced secondary HLH during and post-infection have been sparsely reported [10-15]. In these case reports, the cause of HLH has been postulated to be secondary to subclinical inflammation and dysregulation of the immune system in patients of a wide age range with PCR-proven recovery [15-17]. At the beginning phase of the severe infectious stage of COVID-19, it is difficult to attribute inflammatory reaction solely to HLH vs. COVID-19 infection without other evidence, especially from a bone marrow biopsy. Due to this conundrum, making a rapid diagnosis of HLH is difficult; therefore, HScore can help predict the likelihood of reactive hemophagocytic syndrome early on [18-20]. In our patient’s case, he had complete resolution of COVID-19 in months with negative PCR results prior to admission with a “sepsis-like” picture with further workup demonstrating a diagnosis of HLH. Conclusive pathophysiology behind post-COVID-19 HLH is yet to be found.

## Conclusions

This case report highlights the importance of maintaining a high index of suspicion for HLH in patients with no clear etiology for multisystem disease. Additionally, we wanted to emphasize the seriousness of a history of COVID-19 infection. COVID-19 has been associated with a post-COVID syndrome, and HLH may be one such complication that can result from a history of prior COVID-19 infections.
